# ‘Too young to sit at home’: a qualitative study conducted among employees with young-onset dementia and their relatives

**DOI:** 10.1080/13607863.2024.2345132

**Published:** 2024-04-26

**Authors:** Bo Smeets, Niels Janssen, Kirsten Peetoom, Lizzy Boots, Christian Bakker, Marjolein de Vugt

**Affiliations:** a Department of Psychiatry and Neuropsychology/Alzheimer Center Limburg, Mental Health and Neuroscience Research Institute, Maastricht University, Maastricht, The Netherlands; b Radboudumc Alzheimer Center, Nijmegen, The Netherlands; c Department of Primary and Community Care, Radboud University Medical Center, Nijmegen, The Netherlands; d Groenhuysen, Center for Specialized Geriatric Care, Roosendaal, The Netherlands

**Keywords:** Young-onset dementia, work, continued employment, perspectives, support needs, employees with dementia, relatives

## Abstract

**Objectives:**

Young-onset dementia (YOD) symptoms often first present in the workplace, resulting in work performance challenges and eventually loss of employment. This study aims to investigate the experiences, work values, and support needs of employees with YOD and their relatives.

**Method:**

Semi-structured interviews were conducted to explore the experiences, work values, and support needs of (former) employees with YOD and their relatives. Subsequently, separate focus group discussions were conducted for employees and relatives to review and prioritize interview findings. Inductive thematic analysis was applied to both datasets.

**Results:**

A total of 15 interviews (six employees; nine relatives) and four focus group discussions (ten employees; six relatives) were conducted. Six themes emerged, with five revolving around the central theme: *desire to work*. The other themes represent essential contributing factors, including *importance of receiving a YOD diagnosis; knowledge, awareness, and understanding regarding YOD; open communication, joint decision making, and collaboration; work adjustments, involvement, and support; phasing out work and future perspectives.*

**Conclusion:**

The findings highlight a strong desire to work post-YOD diagnosis, serving as a foundation for developing workplace support tools and guidance. This has the potential to help individuals with YOD preserve their self-confidence and identity while working within their capabilities.

## Introduction

Young-onset dementia (YOD) is characterized by symptom onset before the age of 65 years (van de Veen et al., 2021). Currently, it is estimated that approximately 3.9 million people worldwide are living with YOD. In the Netherlands, the prevalence is estimated to be 14,000 to 17,000 people with YOD (Hendriks et al., 2021). People with YOD are often in an active stage of life, still engaging in work and social activities at the time of disease onset (Giebel, 2021; Pipon-Young et al., 2011).

The onset of dementia symptoms is often insidious and noticed for the first time in the workplace (Harris & Keady, 2009). Symptoms often comprise not only cognitive symptoms but also affective, behavioural, or social symptoms, including personality changes, apraxia/visuospatial dysfunction, memory issues, and language impairment. These symptoms typically develop years before a formal dementia diagnosis is made (Draper & Withall, 2016; Hendriks et al., 2022; Koedam et al., 2010; van Vliet et al., 2013). In the workplace, these symptoms may result in employees having difficulties executing day-to-day tasks and planning, experiencing overall dysfunction, and receiving poor assessments (Chaplin & Davidson, 2016; Evans, 2019; Öhman et al., 2001). Additionally, early symptoms of YOD are frequently misattributed and challenging to identify, as they overlap with symptoms of other conditions, such as burn-out and depression, which contributes to a delay in diagnosis (Loi et al., 2020; Mendez, 2006; O’Malley, Parkes, et al., 2019). In most cases, these symptoms result in sickness absence and a loss of employment (Sakata & Okumura, 2017). Loss of employment has a great impact on both the employee with YOD and their families in terms of financial, psychological, and relational changes (Van Vliet et al., 2011; Werner et al., 2009).

Previous research indicates that following a YOD diagnosis, engaging in daily life activities, such as work, provides structure in daily life and a sense of purpose and potentially enhances perceived quality of life (Roach & Drummond, 2014; Robertson & Evans, 2015). In addition, this engagement facilitates the preservation of an active role in society, which may help individuals maintain their self-esteem, self-confidence, and identity (Harris & Keady, 2009; Phinney et al., 2007). The significance of work extends beyond mere employment, encompassing aspects such as social connections, job satisfaction, and daily routine. However, not every individual with YOD may desire to continue working; therefore, their wishes and needs regarding continued employment should be identified and prioritized.

If continued employment is desired, appropriate support needs to be in place to help employees with YOD in the workplace, meet their needs and focus on their capabilities (Ritchie et al., 2017; Silvaggi et al., 2020). Only a few studies have focused on continued paid employment and, more particularly, on the experiences of employees with YOD and the impact of the condition on their work performance (Andrew et al., 2018; Ikeuchi et al., 2020; Nygard et al., 2023; Omote et al., 2023; Ritchie et al., 2020). Overall, findings from these studies underline the importance of increased awareness and understanding regarding YOD and the possibilities for continued employment. However, clear guidance on how to support employees with YOD in continuing paid employment is still lacking. Examining the perspectives of employees with dementia, relatives of these employees, and employers is paramount and would provide a comprehensive understanding of the topic by integrating personal experiences, caregiving challenges, and workplace dynamics, which would enhance the development of tailored support.

Given the limited research on supporting employees with YOD during their employment, it is important to gain more insight into the experiences, (work)values, and support needs of employees with YOD, including the perspective of their relatives. These insights can serve as a foundation for the development of practical tools and guidance to facilitate and support continued employment for individuals with YOD. This study therefore aimed to explore the experiences, (work)values, and support needs of (former) employees with YOD and relatives of (former) employees with YOD living in the Netherlands.

## Methods

The study presented here is part of the Dutch Work with DEMentia (WorkDEM) project. The project aims to develop practical tools and guidance for continued employment for individuals with YOD based on their specific needs and capabilities. In this qualitative study, semistructured individual interviews and subsequent focus group discussions were conducted with (former) employees with YOD and relatives of (former) employees with YOD, hereafter referred to as employees and relatives.

These interviews aimed to gain insight into the experiences, (work)values and support needs of employees with YOD regarding continued employment. Semistructured interviews were chosen for their ability to explore motivations, choices, experiences, and attitudes regarding a specific topic (Newcomer et al., 2015). Furthermore, they allow for the inclusion of additional questions and in-depth elaboration beyond the established topic list (Galletta, 2013). The focus group discussions reflected on the findings of the previous individual interviews and were used to exchange ideas on possible practical solutions regarding continued employment for employees with YOD. In addition, focus group discussions were selected for their capacity to acquire a comprehensive understanding of diverse perspectives on the given topic and to investigate the dynamics among the participants (Parker & Tritter, 2006).

### Participants and recruitment procedure

For the individual interviews, the aim was to include three to five participants per identified target group (i.e. employees with dementia and relatives). For the focus group discussions, the aim was to organize one focus group discussion per target group including three to six participants (Raats, 2019). The small sample size of the focus groups allowed us to explore complex, emotional topics (Litosseliti, 2003). Regarding employees, the inclusion criteria comprised 1) having an established dementia diagnosis, 2) being (self)employed or having recently ceased working (up to two years previously), and 3) residing in their own household. Inclusion criteria for relatives comprised 1) being at least 18 years old and 2) having a relationship with an employee with YOD (e.g. partner or son/daughter).

A purposive sampling method was applied due to the need for in-depth information regarding the experiences, values and support needs of the target groups involved (Palinkas et al., 2013). The selection of individuals was based on their first-hand experiences with employment and YOD as either an employee with YOD or a relative of such an employee. Participants were primarily recruited *via* the network of involved project partners through, for example, social media and personal approaches. Additionally, flyers were distributed at several memory clinics and YOD care facilities throughout the Netherlands. Participants who participated in the individual interviews were also invited to participate in the focus group discussions.

When individuals were interested in participating, they could contact the researcher (BS) for more information. All interested individuals received an information letter and had to sign an informed consent form prior to participating in the online interviews. Anonymity was ensured by anonymizing the entire process and removing all personally identifiable information.

### Data collection and procedures

Individual interviews took place between May and September 2022. These interviews were guided by a topic list created by the research team and with the input of the project partners, which consisted of employers, an occupational physician, dementia care professionals, patient and care organizations, and the expertise centre for YOD. A topic list was used to allow for additional questions and further elaboration on the topics (Galletta, 2013). The following topics were covered in both target groups: 1) work experiences before the diagnosis of dementia; 2) working with dementia; and 3) support in the workplace after the diagnosis of dementia. This list was subsequently tailored to the employees and relatives. Each interview lasted approximately 45 to 90 min and was audio recorded. The interviews were mainly conducted online *via* Zoom due to the COVID-19 situation at that time and for convenience since the participants’ residences were spread across the Netherlands. Participants received a digital manual on how to use Zoom beforehand. During the interviews, employees were permitted to have their relative accompany them to provide comfort and assistance in setting up Zoom. Participants were invited to read the interview summary for verification purposes. However, no changes were made by the participants. This member check procedure enhanced the quality of the data obtained from the interviews (Thomas, 2016).

Focus group discussions took place between December 2022 and February 2023 and were also conducted online. Each focus group discussion was video recorded and lasted between 90 and 120 min. Prior to the focus group discussion, participants received a factsheet of the individual interviews and filled out a booklet with questions regarding work and YOD. This context mapping sensitized the participants regarding this topic beforehand (Visser et al., 2005). Each focus group discussion was moderated by an experienced moderator (LB/KP). In addition, another researcher (BS) made field notes and observations during these discussions and monitored the whole process. The topic list was based on the topic list used in the individual interviews and was reviewed by different researchers (MdV, CB, KP, BS) with different backgrounds. This list comprised the following topics: 1) reflecting on the findings from the individual interviews using the fact sheet; 2) possible solutions to support an employee with YOD in the workplace; and 3) defining outcome measures regarding the evaluation of these solutions. The questions were tailored to the different target groups. Participants were invited to read the interview summary for verification purposes.

### Data analysis and trustworthiness

Recordings of the individual interviews and focus group discussions were transcribed verbatim, and an inductive thematic analysis was conducted (Braun & Clarke, 2006). As a first step, the transcripts of the individual interviews were analysed. To extract relevant themes from the data and ensure trustworthiness, two independent researchers (NJ and BS) first openly coded the data using Atlas.TI (Elo & Kyngäs, 2008). Thereafter, the researchers reviewed and compared their transcripts and codes and discussed their codes. Subsequently, the codes were organized into different categories and discussed thereafter to reach consensus on the themes. These themes were subsequently discussed with a third researcher (KP). Then, themes were discussed during team meetings with three other researchers from different backgrounds: two health care psychologists and one health care scientist with expertise in YOD (MdV, CB, KP). Subsequently, the focus group transcripts were independently analysed by the same two researchers. The interview data codes were used deductively to analyse the focus group transcripts, identifying new themes to complement the interview data and verify the accuracy of the previously analysed interview data. Thereafter, all identified themes were further discussed in team meetings involving the other researchers (MdV, CB, and KP) to achieve consensus on the final themes retrieved from both interview and focus group data. This process further eliminated potential researcher bias (Norris, 1997) and ensured investigator triangulation (Carter et al., 2014).

Additionally, method and data source triangulation were ensured by using both interviews and focus group discussions as methods, different data sources, such as audio and video recordings and field notes, and context mapping through a summary including a booklet with sensitizing questions (Carter et al., 2014; Creswell, 2013). This study followed the criteria of the Consolidated Criteria for Reporting Qualitative Research (COREQ) to ensure sound qualitative research, rigorous methodology and transparent reporting (Tong et al., 2007).

### Ethical considerations

Ethical approval was obtained from the Medical Ethics Review Committee (METC) of Maastricht UMC+ (azM/UM) for both the individual interviews and focus group discussions (METC 2021-3052 and METC 2022-3267).

## Results

### Participant characteristics

In total, 15 individual interviews were conducted, with six employees and nine relatives (Table 1). Subsequently, four focus group discussions were held with 16 participants, including 10 employees and six relatives (Table 2), four of whom also participated in the individual interviews. The mean age of the employees was comparable in the interviews and focus groups. Additionally, more male employees participated in the interviews than female employees. The sex distribution was equal for the focus group discussions. Overall, a diagnosis of Alzheimer’s disease was reported most often, followed by Lewy body dementia.

**Table 1.** Characteristics of (former) employees participating in the individual interviews and focus group discussions.

Variable	Individual interviews (n = 6)	Focus group discussion (n = 10)
Sex Male (%) Female (%)	5 (83.3) 1 (16.7)	5 (50.0) 5 (50.0)
Age* Mean (min–max)	60.5 (55–65)	59.1 (54–65)*
Diagnosis Alzheimer’s disease (%) Lewy body dementia (%) Frontotemporal dementia (%) Vascular dementia (%)	2 (33.3) 3 (50.0) 1 (16.7) −	8 (80.0) − 1 (10.0) 1 (10.0)

* Age at the time of the interview; missing for three persons.

**Table 2.** Characteristics of relatives participating in the individual interviews and focus group discussions.

Variable	Individual interviews (n = 9)	Focus group discussion (n = 6)
Sex of the relative Male (%) Female (%)	6 (66.7) 3 (33.3)	0 (00.0) 6 (100.0)
Sex of their relative with YOD Male (%) Female (%)	5 (55.6) 4 (44.4)	5 (83.3) 1 (16.7)
Age of their relative with YOD* Mean (min–max)	60.3 (54–67)	N/A
Diagnosis of their relative with YOD Alzheimer’s disease (%) Lewy body dementia (%) Frontotemporal dementia (%) Vascular dementia (%) Mixed dementia (%)	3 (33.3) 2 (22.2) 3 (33.3) − 1 (11.2)	4 (66.7) 1 (16.6) 1 (16.7) - -
Relationship to relative with YOD Partner/Spouse Child Child-in-law Sibling	4 (44.4) 3 (33.3) 1 (11.1) 1 (11.1)	4 (66.7) 2 (33.3) - -

* Age at the time of the interview; missing for one person.

For relatives, a higher percentage of men participated (66.7%) in the individual interviews, whereas in the focus group discussions, only women participated. The sex of the relative with YOD was more equally distributed in the individual interviews than in the focus group discussions. Overall, relatives were mainly the partner/spouse of the person with dementia. The most common diagnosis of their relative with YOD was Alzheimer’s disease, followed by frontotemporal dementia.

### Themes regarding continued employment after YOD diagnosis from the perspective of employees and relatives

**Figure 1.**
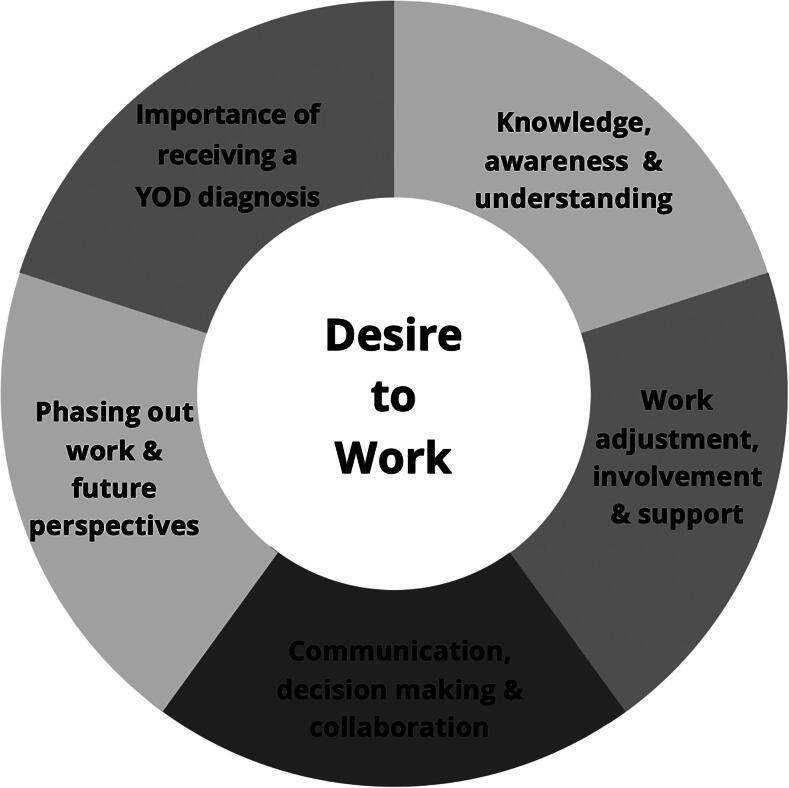
Themes derived from the individual interviews and focus group discussions regarding continued employment after YOD diagnosis.

Six themes were derived from the interview and focus group data and are shown in Figure 1. Of these six themes, five themes were centred around the main theme, *Desire to work*. The other five themes were important conditional factors contributing to the desire to work, including *2) importance of receiving a YOD diagnosis, 3) knowledge, awareness, and understanding of YOD, 4) open communication, joint decision-making, and collaboration, 5) work adjustments, involvement, and support, and 6) phasing out work and future perspectives.*

#### Desire to work

Employees with YOD highlighted a strong desire to work and continue their employment in their own workplace. They stated that work provided them with a sense of self and feeling useful and that they were still able to contribute to society. In addition, work provided them with a daily structure, and it ensured social contact with others. Employees stated that there were still many things they could do in the workplace, such as more structured or adjusted tasks. According to both the employees and relatives, life, especially work life, should not end with a dementia diagnosis.

And not for the money, but for your self-image as well – Employee (EwD006), male, individual interview

Yes, you feel valued [when you keep working]. You feel that you matter. Then, you think, ‘hey, luckily, I still belong somewhere.’ That’s the feeling that is really nice for me. – Employee (EwD701), female, focus group discussion

Relatives also emphasized the importance of work for their loved one. They frequently mentioned that their loved one still had a desire to continue their work.

So that [work] was also a bit of her outlet. There [at work], she could take care of people, and yes, she missed that a lot. I’m 100% certain of that, she said so too. – Relative (R106), male, individual interview

Relatives also highlighted the importance of a daily structure and participation in society through employment.

You are sticking to your routines longer (due to work) […] Suddenly that’s over, and you end up sitting at home. That becomes a burden for everyone in the family, but certainly for the person concerned: what are you going to do all day? – Relative (R104), male, individual interview

#### The importance of receiving a YOD diagnosis

##### Importance of a timely and accurate diagnosis

Participants emphasized the importance of a timely and accurate diagnosis to facilitate their desire to work. They noted significant delays, with some receiving incorrect diagnoses like burn-out or depression, further prolonging the diagnostic trajectory. This uncertainty led to tensions and misunderstandings in the workplace. Consequently, employees faced challenges such as being less efficient, reduced task performance, and conflicts with colleagues or managers.

It was harder to finish, to complete tasks. So, I then tried to pass those off to a colleague. – Employee (EwD003), male, individual interview

I believe that [the conflict with her manager] happened in 2019 or 2018; she got a new manager. She was pretty upset about that because that person didn’t like her, at least according to my sister. It even led to verbal tirades at work. – Relative (R101), female, individual interview

The difficulties surrounding the diagnostic trajectory significantly affected employees, resulting in prolonged sick leave or the risk of job loss due to declined work performance. This sometimes led to ineligibility for disability benefits or dismissal, causing significant and often irreversible financial consequences. Timely and accurate diagnosis could have prevented many negative work-related events, according to employees and their relatives. They noted that as the diagnosis remained uncertain, it became increasingly difficult to maintain paid employment as the disease progressed and work performance deteriorated.

I was just fired, got a commutation, but it was not clear then that I had dementia, no. That came later. – Employee (EwD603), male, focus group discussion

##### ‘Something feels off’ and the role of the general practitioner

Relatives indicated that they often had a strong feeling that ‘something was off’ with their loved one for a long period before a clear diagnosis had been established. Relatives reported that it was difficult to put their finger on what exactly was going on. This led to discussions and tension in the relationship.

Then, I got a kind of gut feeling, which was years back in 2016, that something was not right. From work, they thought he was suffering from burnout symptoms. However, I thought, something else is going on. – Relative (R809), female, focus group discussion

Relatives frequently raised their concerns with their GPs and felt the GP plays a crucial role in early recognition of possible dementia. However, some relatives felt their GP did not take them seriously, contributing to the diagnostic delay.

The GP kept brushing it off, and I was told to send her along by herself. She visited 10 times a year. She would forget the next appointment, and I had to call the GP again and I said: haven’t you noticed? The GP responded with “Yes, yes, everyone forgets something sometimes.” – Relative (R107), male, individual interview

On the other hand, some relatives indicated that their GPs were attentive and took them seriously. This resulted in a timely response to the concerns of the relatives, which allowed timely action to establish a diagnosis.

We [her relatives] went to her GP. We told our story and what we noticed. Well, she also thought that was quite alarming, also because it runs in the family. – Relative (R101), female, individual interview

In some cases, the employees stated that they had a strange feeling about themselves and took the initiative to visit their GP.

But for myself, I did feel that something wasn’t right, and then I went to the GP, and that’s when, you could say, the whole process was initiated to get the diagnosis. – Employee, (EwD001), male, individual interview

##### Importance of a diagnosis for the work environment

All participants stressed that sharing the diagnosis helped clarify changes in work performance or behaviour, fostering understanding and empathy among employers and colleagues. In many instances, this also facilitated the necessary work adjustments to allow them to remain in the workplace for a longer period.

I told my employer, “Yes, there’s something not right, and I’m going to see the doctor.” Because I was so open about it, in the beginning, my employer was very supportive and regularly inquired about how things were going during scans and examinations. So, that actually created an even stronger bond. – Employee (EwD001), male, individual interview

#### Knowledge, awareness, and understanding of YOD

Participants highlighted a perceived lack of awareness among GPs, occupational physicians, employers, colleagues, friends, and family members, therefore emphasizing the importance of knowledge about YOD symptoms, consequences, and its impact on employment. This lack of awareness often led to misunderstandings about the decline in functioning at home and work, as well as delayed or incorrect diagnoses and inadequate workplace support. Additionally, both groups experienced stigma associated with YOD, with employees feeling treated differently, particularly when the diagnosis was unclear.

Late-onset dementia, most people know what it is. However, for young-onset dementia, many are unaware. – Employee (EwD002), female, individual interview

Around a year before the diagnosis was made, he started making more and more mistakes. In the end, he did have a conflict with the supervisor there. Because, well, they thought he was displaying somewhat strange behaviour. – Relative (R103), female, individual interview

#### Open communication, joint decision-making, and collaboration

Both employees and relatives expressed the importance of open communication about the diagnosis in the work environment. Open communication enabled a better understanding of their situations. Moreover, it facilitated the exploration of workplace adjustments to maintain employment, if desired and possible for the employee.

For me, the most important thing is openness about the diagnosis. I notice that I often still encounter a lot of misunderstanding from people, not only specifically about me but also when it concerns others [with YOD]. – Employee (EwD503), female, focus group discussion

Moreover, both employees and relatives stressed the importance of regular communication in the workplace regarding the employee’s work performance, work adjustments and ability to continue employment. In most cases, this led to continued employment for a certain period, e.g. months or a few years. Additionally, it facilitated the exploration of options should work no longer be possible and how to provide for a smooth transition out of their working life.

That [understanding] is a key point that all organizations have to deal with, and if there is understanding, including from the employer, then you can achieve many more of those other points [continue employment within the workplace] – Employee (EwD501), male, focus group discussion

Regular meetings between employee, employer, and other relevant stakeholders, to discuss the wishes, needs, and capabilities of both parties regarding facilitation of arrangements for continued employment, helped. This allowed for early adjustments to work tasks or exploration of alternative options, such as gradual retirement or cessation of work. Joint decision-making empowered employees, fostering a sense of control over their lives. As a result, employees felt heard, engaged, and in control within the workplace.

My supervisor regularly contacted me to see how I was doing and to assess my situation. He would inquire whether it would be wiser to work less due to the increasing workload. So, through frequent communication, he provided me with good support, which also strengthened our relationship. – Employee (EwD001), male, individual interview

Collaboration is essential, but so is full recognition. It’s important that the person with Alzheimer’s treats themselves as a whole person and that others do the same. Everyone should be in an equal position regarding being treated with dignity, just as they were before. – Employee (EwD702), male, focus group discussion

##### Collaboration among professionals

Relatives mentioned the importance of involving relevant professionals, such as the GP, occupational physician, and dementia case manager, especially regarding receiving a timely diagnosis and continuing employment. Establishing collaboration between the care environment and the work environment may result in a better understanding and facilitate continued employment if desired.

When employers and health care professionals collaborate, it makes a huge difference. – Relative (R808), female, focus group discussion

However, employees along with relatives had varying experiences with the involvement of these professionals. Some experienced the involvement sufficient, while others felt it was lacking. Consequently, unwanted scenarios arose, such as dismissal, which could have been avoided by timely engagement of the relevant professionals.

I think the occupational physician could play a significant role. In my case, I believe he did well. They could probably provide more guidance on what is feasible and how to handle things that may no longer be good enough. This way, you can have an active conversation about what you can and cannot do. – Employee (EwD705), male, focus group discussion

#### Work adjustments, involvement, and support

##### Work adjustments and support for employees

In addition to engaging the right professionals and fostering collaboration among them, both employees and relatives highlighted that with suitable workplace adjustments and support, employees with YOD might continue their work for an extended period. Employees’ capabilities and wishes should be used as a starting point, and the focus should be on what employees with YOD can still do rather than on what they can no longer do.

They should just let you do it yourself, not take it over from you, or think on your behalf. Just let you do it, maybe you’re slower, but you can still do it. In addition, if you keep doing it, they will see that you can still handle everything. Often, they say: don’t do too much, be careful, don’t do this.’ It’s really patronizing to the extreme. – Employee (EwD501), female, focus group discussion

It’s not about what you can no longer do, as we all know that. It’s about focusing on what you can still do. – Employee (EwD002), female, individual interview

Both employees and relatives mentioned examples of work adjustments and support, including task reduction, reduced working hours, establishing clear boundaries and structure, receiving assistance from colleagues, and creating a low-stimulus work environment. However, they noted that the feasibility of these adjustments depends highly on the type of work, as well as the level of responsibility and independence required.

##### Involvement of relatives

Relatives expressed a need for greater involvement in the process of continued employment from their loved one, both during declines in work performance and after the YOD diagnosis. They believed that early involvement could have facilitated a more timely diagnosis by combining perspectives from both home and work environments. However, privacy regulations were considered a barrier, hindering them to openly communicate with employers or occupational physicians.

What I really missed is the involvement with the employer, that they didn’t engage in a conversation with him about: ‘gosh, how come you’ve functioned well for 23 years?’ He was also a project manager where he handled heavy projects; ‘how come you can’t keep up with this now?’ There’s a piece in there that I really miss. I would have liked to be included in that. – Relative (R802), female, focus group discussion

After the dementia diagnosis, relatives indicated a wish to be involved regularly to evaluate whether everything was still going well or if adjustments or support changes needed to be made at work. They also wished to be more involved in conversations with the occupational physician about the possibilities regarding the continued employment of their loved one.

What I needed was a form of collaboration, where we could create a sort of sustainability together. A network where we could support each other, have a conversation about what we noticed and find ways to address it. – Relative (R108), female, individual interview

##### Support for relatives

Relatives indicated specific support needs to aid their loved ones in continue working, seeking greater flexibility and freedom in their own workplaces to take time off when necessary. Additionally, they felt that dementia case managers should be involved more timely and could play an important role. For instance, by informing the employer about dementia and how a person with dementia can be supported in the workplace. Furthermore, they sought additional support and understanding from various professionals in both the workplace and caregiving roles involved in their loved one’s employment process.

Not having to go through all sorts of obstacles. That’s one. The strongest obstacle you have now, of course, is the diagnosis. But being able to get help earlier, and I also think, this is quite broad, but support with arranging everything and all that is available. Assistance in everything – Relative (R808), female, focus group discussion

#### Phasing out work and future perspectives

##### Phasing out work

Although most employees indicated a desire to work for as long as possible, it is inevitable that there comes a point in time when continuing to work is no longer feasible. Employees stated that when they were involved in the decision-making process of phasing out work, it became easier to accept that their working life phase was coming to an end. This involvement also helped them come to terms with the diagnosis and its future implications. Additionally, they noted that their involvement in the decision-making process about winding down or phasing out work also facilitated them to accept that work was no longer possible.

And that, in that way, also makes it easier for me to accept. You can now see it, like; ‘Yes, it really can’t work anymore, it’s truly not possible.’ How on earth can I help at the [care institution] if I can’t perform my tasks properly. – Employee (EwD701), female, focus group

Employees also indicated that they still felt a great responsibility and often struggled to delegate their work to others. Involving them in this process and winding down at work step-by-step gave them a more reassurance.

Well, sometimes it feels like I’m losing my job, like I’m training someone else to take over. – Employee (EwD005), male, individual interview

And at some point, it just doesn’t work anymore, and you have to come to terms with that. It took quite a while, about two or three months, because I went on sick leave in October, and I think it was in February when my occupational physician asked me if I was ready to leave the working life behind. I said: ‘Yes, now I am.’ Because I wasn’t ready before, partly due to the connection we [the employer and employee] had with each other. – Employee (EwD604), female, focus group discussion

##### Future perspectives

Employees as well as relatives indicated that it was important that the transition between work and postwork life was as smooth as possible. A proper farewell to their employer and colleagues contributed to this smooth transition.

I think the most important thing is that I can still have this destination and see these lovely people and have a nice closure. – Employee (EwD003), male, individual interview

Furthermore, employees mentioned that they viewed themselves too young to be idle all day. They still desired to have had some kind of structure in their life. Their ability to sustain a sense of purpose in life and perceive themselves as making meaningful contributions to society was equally important.

It’s very challenging because you were always working, and it was responsible work since you were alone in those households. Now, being at home, it’s tough. You don’t have a routine… you really have to create a schedule for yourself, or else you feel completely disconnected from people. – Employee (EwD701), female, focus group discussion

Both employees and relatives also perceived the use of day care as too early after their working life had come to an end. Employees indicated that they could still do other things, for example, voluntary activities. However, suitable options for a daily structure after work were very scarce and often did not match the needs of the employees.

I had the same feeling too… I saw those people, you know, and it sounds strange, but I just thought: I’m still way too capable for something like that [day care facility], you know? I shouldn’t do it because it will make me decline further instead of… – Employee (EwD004), male, individual interview

## Discussion

In the present study, we investigated the experiences, work values, and support needs of (former) employees with YOD and their relatives living in the Netherlands. Among the six identified themes, the central theme of the *desire to work* emerged. This overarching theme highlights the strong desire of employees with YOD to actively participate in society through continued employment. The other themes—*the importance of receiving a YOD diagnosis; knowledge, awareness, and understanding of YOD; open communication, joint decision-making, and collaboration; work adjustments, involvement, and support; and phasing out work and future perspectives*—are centred around this overarching theme and are conditional for sustaining continued employment after a YOD diagnosis and fulfilling employees’ desires to work. It is important to note that these themes are not isolated but intricately interconnected.

The theme of *the importance of receiving a YOD diagnosis* is one of the initial aspects that employees encounter. In the Netherlands, GPs are often the first health care professionals approached when individuals face difficulties in daily life. However, GPs may find it challenging to recognize early-stage YOD symptoms due to, for example, their limited exposure to such cases in their careers. This in turn might hamper a timely YOD diagnosis (Hendriks et al., 2022), due to, for example, the misattribution of symptoms (Draper et al., 2016; O’Malley, Carter, et al., 2019), as mentioned in our study. A timely and accurate diagnosis stands as a critical starting point regarding receiving appropriate support if continued employment is desired (Ritchie et al., 2017).

*Knowledge, awareness, and understanding of YOD* were perceived as lacking in this study among both work and health care professionals, including GPs. Additionally, this lack was evident within the social and work environments of the employees with YOD. Previous research has also emphasized this lack (Bruinsma et al., 2020; Ikeuchi et al., 2020; Rabanal et al., 2018). Moreover, the provision of appropriate support and utilization of services by individuals with YOD and their relatives fall behind because of this (Couzner et al., 2022). This might have a negative impact, such as imposing an excessive burden on caregivers (van Vliet et al., 2010). Our study further highlights that the same applies for continued employment, in terms of delayed workplace recognition and often a lack of adequate support measures for both employees and relatives.

The themes regarding *open communication, joint decision-making, and collaboration and work adjustments, involvement, and support* are closely interconnected. Moreover, open communication about the diagnosis is important, as it increases understanding and lays the foundation for support within the work environment. In line with our findings, a recent Swedish study (Nygard et al., 2023) also highlighted the difficulty of recognizing dementia in the workplace and emphasizes the importance of openness about the diagnosis within the work environment. Fostering open communication and collaboration among employees with YOD and their relatives, the work environment (i.e. employer and colleagues), and the health care and work professionals involved is of paramount importance for enacting necessary workplace adjustments and ensuring consistent support throughout the entire diagnostic process (Omote et al., 2023). Furthermore, our study highlights the necessity for relatives to have a more active role in this communication and collaboration. However, current privacy regulations impede this involvement. Last, it is worth noting that dementia can manifest in various ways, resulting in different disease trajectories that may influence the feasibility of continued employment (Gerritsen et al., 2018).

This study also emphasizes that *phasing out work and future perspectives,* was important to the participants since not all employees with YOD wish to continue working. Instead, they prefer a seamless transition from work to postwork life, involving careful planning with professionals, such as dementia case managers, regarding future activities. Previous studies have shown that being active and being able to contribute to society is highly beneficial and might have positive effects on the disease course as well (Hewitt et al., 2013; Richardson et al., 2016). On the other hand, it is important to note that a certain level of disease insight is necessary to engage in open dialogue and make effective agreements regarding potential work adjustments or phasing out work. However, limited disease insight, observed for forms such as frontotemporal dementia (FTD), might hinder this engagement (Kurz et al., 2014). In addition, not all professions are suited for work adjustments to the extent that continued employment remains feasible, e.g. professions with high levels of autonomy and responsibility.

As demonstrated by our study, the necessity for tailored workplace support or the exploration of alternative options outside paid work that offer structure, safety, dignity, a sense of purpose, and societal contribution for individuals with YOD is emphasized. This study therefore highlights the need for enhanced alignment and support for employees with YOD and their work environment. Nonetheless, concrete support measures or interventions within the workplace context for YOD are still scarce, representing a significant gap in the field.

### Implications for practice and future research

Findings from this study not only hold significance for the work environments of individuals with YOD but they resonate also at a broader policy and societal level. The results have the potential to catalyse positive changes, influencing workplaces, policies, and societal perspectives regarding YOD. Additionally, while the setting for (continued) employment may vary across countries due to differences in laws, regulations, and the organization of employment, the outcomes of this study hold relevance beyond the Dutch context. This is because aspects of this study results such as knowledge, awareness, communication and collaboration, participation in society, and preservation of identity are not constrained by context. They are fundamental values intrinsic to individuals with YOD. Therefore, these findings can be applied across different contexts and are not limited to the specifics of the Dutch context.

Furthermore, this study stresses that the desire to work and to continue employment is not a societal aspect to be overlooked but rather a fundamental aspect that demands acknowledgement and proactive support. In education and awareness efforts, it is essential to devise strategies to provide this support in addition to addressing the needs and capabilities of employees with YOD. To achieve this, launching a public awareness campaign focused on this theme could be essential, aligning well with the goal of creating a dementia-friendly society by raising awareness and providing education about YOD and subsequently reducing stigma (Hung et al., 2021). Also, early family involvement aids in timely diagnosis, while open communication and collaboration among stakeholders, including relatives, employers, occupational physicians, and health care professionals, are essential for continued employment. Furthermore, when the workplace process operates more smoothly, individuals are able to continue their employment in alignment with their preferences and capabilities. This, in turn, facilitates a smoother transition when phasing out work. This will benefit not only employee well-being but also the surrounding environment. Furthermore, it is imperative for the government to play a facilitating role in this whole process, as it oversees rules and regulations that, in some areas, create barriers in taking necessary steps for employees with YOD.

To ensure timely diagnosis and support in the work context, enhancing knowledge and awareness among health care professionals, employers, and society as a whole is important. This can be, for example, established through the WorkDEM project, which aims to develop practical tools to guide and support continued employment for all stakeholders involved in the Netherlands. However, elements of this Dutch project, could also be transferable over the long term to different countries with the necessary context-specific adjustments. In addition to employees with YOD and relatives, future research should include the perspectives of other important target groups, such as employers, occupational physicians, and other relevant health care professionals, to tailor practical tools and guidance to facilitate and support continued employment in the context of YOD. Ultimately, this holistic approach could have a positive impact on living and working well for individuals with YOD, in alignment with the modern concept of inclusion in the workforce beyond paid employment, that acknowledges that individuals with disabilities such as YOD, should have opportunities to contribute to the workforce in various capacities. This concept aligns with disability acts and rights frameworks across various countries that recognize that employment is not only about income but also about fostering dignity, self-worth, and inclusion in society (Fryers, 2006).

### Strengths and limitations

One of the main strengths concerns the inclusion of both employees with YOD and their relatives, as this offers two essential and interlinked perspectives on continued employment. Second, this study included individuals with various common forms of YOD, which differ significantly in their presentation, as well as with different employment backgrounds. Additionally, the topic of this research has not been investigated before in the Netherlands. We aim to address the need for research on this topic through this study.

A further strength concerns the use of method and data source triangulation, combining interviews, focus group discussions, various data sources such as audio and video recordings and field notes, and context mapping. This approach enhanced the study’s credibility and provided a more in-depth understanding of the topic and ensured investigator triangulation (Carter et al., 2014; Creswell, 2013).

A potential limitation regards the requirement for participants to reflect on their work lives and sufficiently express themselves verbally. This could have led to selection bias (Smith & Noble, 2014). Although this study included individuals with various subtypes of dementia and different employment backgrounds, future studies should also include individuals with diverse cultural backgrounds and ethnicities to explore potential variations in dementia perceptions and coping mechanisms concerning topics such as continued employment and YOD.

## Conclusion

To meet the desire of individuals with YOD to work and to provide tailored support in continued employment, it is fundamental to increase awareness, knowledge, and collaboration among work and health care professionals, the work environment, relatives, and employees. Findings from this study will be used as a starting point for the development of practical tools and guidance for adjustments within the work context for YOD (WorkDEM). Focusing on the wishes and needs of employees with YOD is paramount and should form the basis for decisions regarding continued employment and future perspectives. Despite the fact that this study was conducted in The Netherlands the results remain relevant and applicable to other countries and work environments. This universality comes from the fact that the results are rooted in fundamental, universal values shared by people globally. To conclude, there is no one-size-fits-all solution for continued employment, emphasizing the importance of tailored support for employees with YOD within their unique capabilities and circumstances. Acknowledging and addressing these diverse needs in an international context, can foster inclusivity, dignity, and empowerment for individuals with YOD in the global workforce.
